# Free tissue flaps in head and neck reconstruction: clinical application and analysis of 93 patients of a single institution^☆^

**DOI:** 10.1016/j.bjorl.2017.04.009

**Published:** 2017-05-13

**Authors:** Jiwang Liang, Tao Yu, Xu Wang, Yuejiao Zhao, Fengqin Fang, Wei Zeng, Zhendong Li

**Affiliations:** a Cancer Hospital of China Medical University, Liaoning Cancer Hospital & Institute, Department of Head and Neck Surgery, Shenyang, China; b Cancer Hospital of China Medical University, Liaoning Cancer Hospital & Institute, Department of Radiology, Shenyang, China; c Liaohe Oil Field General Hospital, Department of Surgical Oncology, Panjin, China

**Keywords:** Free flaps, Head and neck, Outcomes, Reconstruction, Retalhos livres, Cabeça e pescoço, Resultados, Reconstrução

## Abstract

**Introduction:**

Reconstruction with a free flap is routine in head and neck surgery because of better functional outcomes, improved esthetics, and generally higher success rates.

**Objective:**

To evaluate the clinical outcomes in patients undergoing different microvascular free flap reconstructions.

**Methods:**

This was a retrospective study of 93 patients undergoing reconstructions with free flaps from 2007 to 2015. Four types of free flap were performed: anterolateral thigh (76.3%), radial forearm (16.1%), fibula (4.3%) and jejunum (3.3%). Patients’ demographic data were collected, and the outcomes measured included flap survival and complications. Postoperative functional and oncological outcome were also analyzed.

**Results:**

The patients included 73 men and 20 women, with a mean age of 56.1 years. The most common tumor location was the tongue. Squamous cell carcinoma represented the vast majority of the diagnosed tumors (89.2%). The most common recipient vessels were the superior thyroid artery (77.4%) and the internal jugular vein (91.4%). Nine patients required emergency surgical re-exploration and the overall flap success rate was 90.3%. Venous thrombosis was the most common cause for re-exploration. Other complications included wound infection (5.4%), wound dehiscence (1.1%), partial flap necrosis (9.7%), fistula formation (10.8%), and 1 bleeding (1.1%). The majority of patients had satisfactory cosmetic and functional results of both donor site and recipient site after 46.7 months of mean follow-up.

**Conclusion:**

Microsurgical free flap is shown to be a valuable and reliable method in head and neck surgery. It can be used safely and effectively with minimal morbidity in selected patients. The reconstruction can be performed by appropriately skilled surgeons with acceptable outcomes. Success rate appears to increase as clinical experience is gained.

## Resumo

**Introdução:**

Reconstrução com retalhos livres é um procedimento de rotina nas cirurgias de cabeça e pescoço devido aos melhores resultados funcionais e estéticos, e taxas de sucesso geralmente maiores.

**Objetivo:**

Avaliar os desfechos clínicos de pacientes submetidos a diferentes reconstruções microvasculares com retalhos livres.

**Método:**

Estudo retrospectivo de 93 pacientes submetidos à reconstruções com retalhos livres, de 2007 a 2015. Foram utilizados quatro tipos de retalho livre: coxa anterolateral (76,3%), antebraço radial (16,1%), fíbula (4,3%) e jejuno (3,3%). Os dados demográficos dos pacientes foram coletados e os parâmetros avaliados incluíram sobrevida e complicações. Os resultados funcionais e oncológicos pós-operatórios também foram analisados.

**Resultados:**

Os pacientes incluíram 73 homens e 20 mulheres, com idade média de 56,1 anos. O local mais comum para o tumor foi a língua. O carcinoma de células escamosas representou a maioria dos tumores diagnosticados (89,2%). As artérias receptoras mais comuns foram a artéria tireóidea superior (77,4%) e a veia jugular interna (91,4%). Nove pacientes necessitaram de reexploração cirúrgica de emergência e a taxa de sucesso global do retalho foi de 90,3%. A trombose venosa foi a causa mais comum da reexploração. Outras complicações incluíram infecção da ferida cirúrgica (5,4%), deiscência da ferida (1,1%), necrose parcial do retalho (9,7%), formação de fístula (10,8%) e sangramento (1,1%). A maioria dos pacientes apresentou resultados estéticos e funcionais satisfatórios, tanto no local doador quanto no receptor, após 46,7 meses de seguimento médio.

**Conclusão:**

O retalho livre microcirúrgico mostrou ser um método valioso e confiável na cirurgia de cabeça e pescoço. Pode ser utilizado de forma segura e eficaz, com morbidade mínima em pacientes selecionados. A reconstrução pode ser realizada por cirurgiões adequadamente qualificados com resultados aceitáveis. A taxa de sucesso parece aumentar à medida que a experiência clínica é adquirida.

## Introduction

Head and neck cancer is the sixth common cause of cancer with an estimated worldwide incidence of over 600,000 new cases annually.^1^ Surgery for tumors of head and neck can cause significant soft tissue, bony and skin defects. This may result in functional impairment such as speech and swallowing deficits. Thus, the reconstruction of extensive defects after resection has always been challenging. In the past, attempts were made to achieve functional restoration of resected head and neck areas with acceptable cosmesis using local and locoregional flaps. The introduction of the Pectoralis Major Myocutaneous (PMMC) flap was well established in 1979 as one of the most important reconstructive methods due to its simple technical aspects and versatility.^2^ However, the major disadvantages were that it was too bulky and the nipple position may become distorted, both of which can cause cosmetic problems. The free flap technique represented a revolution in reconstructive surgery as it enabled the harvesting of a large amount of revascularized tissue, and it could be tailored to the defect and allowed for more complex reconstructive procedures, while simultaneously permitting more extensive head and neck resections.^3^, ^4^, ^5^ Today, microvascular surgery is an essential part of the treatment of head and neck defects.

Different free tissue flaps had been reported in the reconstruction of tumor defects of head and neck region, such as Latissimus Dorsi (LD) flap, Radial Forearm (RF) flap, scapula flap, Anterolateral Thigh (ALT) flap, Jejunum flap, and Rectus Abdominis muscle (RA) flap.^6^, ^7^, ^8^, ^9^ For example, the RF flap was used extensively in China before the year 2007. It is a fasciocutaneous flap elevated from the volar region of the forearm and based on the radial artery and comitant venous pedicle. In spite of its usefulness and feasibility, at the receiving site, harvesting the RF flap implies sacrifice of an important vessel, namely, the radial artery and a high rate of donor site morbidity. The ALT flap is one of the fasciocutaneous flaps in the thigh based on the septocutaneous or musculocutaneous perforators derived from the lateral circumflex femoral artery and its venae comitantes. It has a large and long vascular pedicle, and because of the distance of the donor site from the head and neck, it can easily be harvested with a 2 team approach. It has been widely applied in soft-tissue reconstruction. The aim of this study was to present our experiences with different free flaps for head and neck defects, and to analyze the characteristics of these flaps in comparison with the literature reviews. The advantages of the free tissue flaps, including their versatility and usefulness, were also discussed.

## Methods

In this retrospective analysis, we investigated 93 consecutive patients undergoing microsurgical reconstructions, which were conducted in the Department of Head and Neck Surgery in our hospital between January 2007 and June 2015. Clinicopathologic features and surgical data were included. The medical records of the primary tumor site, flap type, vessels from the donor site, vessels at the recipient site, and complications were analyzed. All subjects gave their informed consent for the study, and the protocol was approved by our institutional review board. Of these patients, 9 cases had undergone former surgical resection. Three patients had received preoperative radiotherapy (40–55 Gy). Eleven patients received postoperative radiotherapy (35–65 Gy).

All surgical procedures were performed with a two team approach. The experienced senior surgeons were responsible for tumor resections, which were performed with or without neck dissections depending on the nodal status. The other team was responsible for reconstruction of the defects, including flap harvesting, and vascular anastomosis. The operative technique has been well described in previous reports.^10^, ^11^, ^12^, ^13^ We made minor modifications in the surgical technique. We resected the tumor at the normal tissue about 2 cm away from the tumor margin. The frozen pathologic diagnosis determined the radical resection area, and then the dimension of free flap was determined. After the surgery, flap monitoring was performed every 30 min during the first 24 h, then every 1 h for the second and third days, and every 24 h for the following days. Dexamethason 10 mg/days was given intravenous injection in the first 3 days, and dextran 40 1000 mL/days was also infused for the first 5 consecutive days. In the case of adverse changes in the free flap, including a change in color to pale or dark, a loss of luster, or a lowering in temperature, an acupuncture bleeding test using needles was done. Exploration and salvage surgery was performed immediately if the flap did not improve during the next 2 h of monitoring.

The patients were followed-up clinically once a month during the first 6 months and every 6 months for the next few years. Ultrasound examination was performed every 3 months postoperatively and CT or MRI was performed every 6 months to examine whether there were any signs of local recurrence. During the follow-up, the cosmetic results were evaluated by clinical examination and the patient's own perception. The esthetic outcome was categorized as satisfied, neutral, and dissatisfied. Diet was assessed by the patient's dietary intake and was classified as tolerating a normal diet, a soft diet, and a liquid diet. The assessment of speech was based on whether the patient could be understood easily by a listener (fluent and intelligible) or whether it required concentration on the part of the listener (intelligible with effort). Speech was judged as unintelligible if the patient could not be understood. All patients were followed after surgery until May 2015. The median follow-up duration was 44.2 months (range, 1.9–100.3 months). Compliance was 96.8%, with only 3 patients lost to follow-up.

All statistical analyses and graphics were performed by SPSS 16.0 statistical package (SPSS, Inc., Chicago, IL, USA). A Chi-square test was used for comparison of categorical variables. A value of *p* < 0.05 was considered statistically significant.

## Results

There were 73 men and 20 women in our series. The mean age of patients was 56.1 years. The patient characteristics are detailed in Table 1. The length of hospitalization ranged from 18 to 105 days, with a mean duration of 36.6 days. The ALT flap was the flap most often harvested in our patients (*n* = 71). The RF flap was the second most used after the ALT flap (*n* = 15), followed by the fibula myocutaneous flap (*n* = 4) and the jejunum flap (*n* = 3). The tongue was the site most commonly involved (*n* = 23), followed by the oropharynx (*n* = 22), hypopharynx (*n* = 17), floor of the mouth (*n* = 11), buccal mucosa (*n* = 10), skin (*n* = 4), sublingual gland (*n* = 2), jaw bone (*n* = 1), lip (*n* = 1), gum (*n* = 1), and larynx (*n* = 1). The histologic diagnosis of the tumors was squamous cell carcinoma (89.2%), adenoid cystic carcinoma (4.3%), adenocarcinoma (2.2%), basal cell carcinoma (1.1%), malignant melanoma (1.1%), mucoepidermoid carcinoma (1.1%), and malignant fibrous histiocytom (1.1%). Clinical staging was performed according to the seventh edition of the UICC/AJCC TNM staging system. The tumor stages were as follows: stage I in 7 patients (7.5%), stage II in 27 patients (29%), stage III in 32 patients (34.5%), and stage IV in 27 patients (29%).

**Table 1:** Clinical data analyses of the patients who underwent free flap for reconstruction.

Variables	Number	Rate (%)
*Gender*		
Male	73	78.5
Female	20	21.5

*Age (years)*		
<55	42	45.2
≥55	51	54.8

*Flap types*		
ALT flap	71	76.3
RF flap	15	16.1

*Fibula myocutaneous flap*	4	4.3
*Jejunum flap*	3	3.3

*TNM classification*		
I + II	34	36.6
III + IV	59	63.4

*Preoperative radiotherapy*		
Yes	3	3.2
No	90	96.8

*Postoperative radiotherapy*		
Yes	11	11.8
No	82	88.2

*Local recurrence*		
Yes	6	6.5
No	87	93.5

*Distant metastasis*		
Yes	18	19.4
No	75	80.6

*Treatment*		
Operation alone	41	44.1
Operation + chemotherapy/radiotherapy	52	55.9

The average ALT flap size was 62 cm^2^ (range, 12–192 cm^2^), the average RF flap size was 42 cm^2^ (range, 12–100 cm^2^), and the average fibula myocutaneous flap size was 32 cm^2^ (range, 12–72 cm^2^). The length of the jejunum flap varied between 12 and 15 cm (mean 14 cm). The mean time taken to harvest the flap was 56.7 ± 9.5 min, while the average time taken to perform the vascular anastomosis was 51.8 ± 6.7 min. Blood transfusion was performed in 53.8% of the cases. Recipient arteries were the following: superior thyroid artery in 72 cases (77.4%), facial artery in 13 cases (14.0%), external carotid artery in 4 cases (4.3%), transversa colli artery in 4 cases (4.3%), and lingual artery in 2 cases (2.2%). The internal jugular vein was the most commonly used recipient vein, and they were used in 85 cases (91.4%). The external jugular vein, facial vein, superior thyroid vein, and retromandibular vein were used in 10 (10.8%), 5 (5.4%), 4 (4.3%) and 1 (1.1%) cases, respectively.

Total flap survival rate was 90.3% (84/93). Nine flaps developed signs of vascular thrombosis postoperatively that necessitated surgical exploration. Three of these flaps were salvageable and 6 flaps were lost. Venous crisis occurred in 5 flaps (55.6%) and the main reason was mechanical obstruction due to thrombosis of vascular anastomosis sites, compression, and twisting. Arterial crisis occurred in 3 flaps (33.3%), and only 1 was salvageable. In one case, the cause of failure was unknown as nothing could be found during re-exploration, despite complete evaluation of the microvascular anastomosis, perforator, and pedicle. It was noted that venous thrombosis was more common than arterial thrombosis. Two flap crises were identified within 16 h after surgery, 4 were identified at 16–24 h after surgery, and 3 were identified > 24 h after surgery. The comprehensive analysis of the patient's medical history has been performed in order to identify the factors that may have the adverse effect on the success rate of the flap (Table 2). No significant differences were found between the two groups in terms of gender, age, smoking and alcohol (all *p* > 0.05). There was no association between postoperative radiotherapy and flap necrosis (*p* = 0.248). Our analysis also indicated lack of association between existence of hypertension or diabetes and increased risk of flap necrosis (*p* = 0.549, *p* = 0.310).

**Table 2:** Clinicopathologic characteristics of 9 patients with flap necrosis.

Variables	Number	Number of flap necrosis	*p*-Value
*Gender*			0.956
Male	73	7 (9.5%)	
Female	20	2 (10.0%)	

*Age (years)*			0.854
≤49	23	2 (8.7%)	
>49	70	7 (10.0%)	

*Postoperative radiotherapy*			0.248
Yes	11	0 (0%)	
No	82	9 (11.0%)	

*Smoking*			0.387
Yes	72	8 (11.1%)	
No	21	1 (4.8%)	

*Alcohol*			0.257
Yes	56	7 (12.5%)	
No	37	2 (5.4%)	

*Hypertension*			0.549
Yes	6	1 (16.7%)	
No	87	8 (9.2%)	

*Diabetes*			0.310
Yes	11	2 (18.2%)	
No	82	7 (8.5%)	

Twenty-six patients (28.0%) developed at least one postoperative complication, including wound infection (*n* = 5), wound dehiscence (*n* = 1), partial flap necrosis (*n* = 9), fistula (*n* = 10), and bleeding (*n* = 1). All these complications were relatively minor, and only one required beside debridement. Despite the fact that many patients had several comorbid conditions and some were obese, there were no serious medical complications or perioperative mortality in the current series.

There were 6 patients of recurrence of the original tumor, and 18 patients had metastasis, including lymph node, lung, cervical esophagus, and parotid gland. Four patients refused treatment, whereas 12 patients were treated by surgical excision and 8 patients received radiotherapy. During the follow-up period, 32 patients die of their disease, 9 are alive with disease (3 local recurrences, 6 distant metastases), and 49 show no evidence of disease. Eighteen patients died of recurrence and metastasis, 5 patients died of myocardial infarction, 5 patients died of mental trauma, one patient died of upper gastrointestinal bleeding, and 3 patients died of unknown causes.

Eighty-six patients (92.5%) participated in the functional outcome analysis. All patients taken an oral diet; 50 (58.1%) of these eat a regular diet, 24 (27.9%) are able to eat soft food, and 12 (14.0%) eat liquid food. As evaluated through conversations with the patients, 46 patients (53.5%) had normal speech, 30 (34.9%) were understood with some abnormality, and 10 (11.6%) were difficult to understand. Majority of patients were comfortable with social interactions. The results of the evaluation of function are listed in Table 3. According to the evaluation, good esthetic results were found in 80.6% of cases. The patients who had postoperative radiotherapy had the poorer esthetic results. Slightly diplopia developed in one patient, but the patient did not complain of any disability in daily life. Four patients complained of mild crust formation, and 6 patients who had postoperative radiotherapy developed moderate crust formation and dryness. With RF flap, two patients develop temporal dysesthesia in the area of the superficial radial nerve, although no patients had definitive dysesthesia in the wrist or hand. No patients complained about movement impairment or donor site weakness of strength in the ALT flap and RF flap, whereas one patient who used fibula myocutaneous flap felt weakness of strength. With jejunum flap, all the patients received total laryngectomy and circumferential pharyngectomy, and none of the patients had fistula during the follow-up period.

**Table 3:** Postoperative function results in all types of head and neck defects.

Type	Number	Long-term complications	Cosmetic appearance	Diet	Speech
ALT flap	66	Recipient site deformity (*n* = 9)	Satisfied (*n* = 38)	Normal (*n* = 39)	Fluent and intelligible (*n* = 37)
		Donor site deformity (*n* = 0)	Neutral (*n* = 20)	Soft (*n* = 18)	Intelligible with effort (*n* = 24)
			Dissatisfied (*n* = 8)	Liquid (*n* = 9)	Unintelligible (*n* = 5)

RF flap	14	Recipient site deformity (*n* = 4)	Satisfied (*n* = 8)	Normal (*n* = 7)	Fluent and intelligible (*n* = 7)
		Donor site deformity (*n* = 0)	Neutral (*n* = 4)	Soft (*n* = 4)	Intelligible with effort (*n* = 5)
			Dissatisfied (*n* = 2)	Liquid (*n* = 3)	Unintelligible (*n* = 2)

Fibula myocutaneous flap	3	Recipient site deformity (*n* = 1)	Satisfied (*n* = 1)	Normal (*n* = 2)	Fluent and intelligible (*n* = 2)
		Donor site deformity (*n* = 0)	Neutral (*n* = 1)	Soft (*n* = 1)	Intelligible with effort (*n* = 1)
			Dissatisfied (*n* = 1)	Liquid (*n* = 0)	Unintelligible (*n* = 0)

Jejunum flap	3	Recipient site deformity (*n* = 2)	Satisfied (*n* = 1)	Normal (*n* = 2)	Fluent and intelligible (*n* = 0)
		Donor site deformity (*n* = 0)	Neutral (*n* = 2)	Soft (*n* = 1)	Intelligible with effort (*n* = 0)
			Dissatisfied (*n* = 0)	Liquid (*n* = 0)	Unintelligible (*n* = 3)

### Case reports

#### Case 1

**Figure 1:**
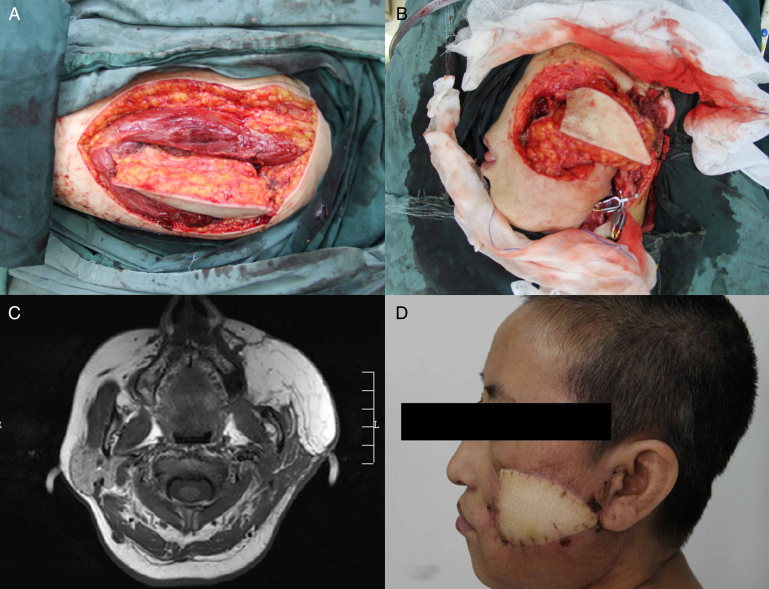
(A) The ALT flap was harvested from the left thigh; (B) the through-and-through defect over left cheek after excision of buccal squamous cell carcinoma; (C) postoperative axial MRI showing the ALT flap; (D) postoperative result 4 months after operation.

A 52 year-old female patient was diagnosed as T_4_N_2_M_0_ squamous cell carcinoma of the left buccal mucosa. After excision of the tumor and radical neck dissection, a 16 cm × 8 cm left ALT flap was harvested.^10^ The flap was folded over to cover both the inner and outer linings. Anastomoses were performed to the superior thyroid artery, the external jugular vein and the internal jugular vein. The postoperative period was uncomplicated. Four months after the operation, the result was satisfactory (Fig. 1A–D).

#### Case 2

**Figure 2:**
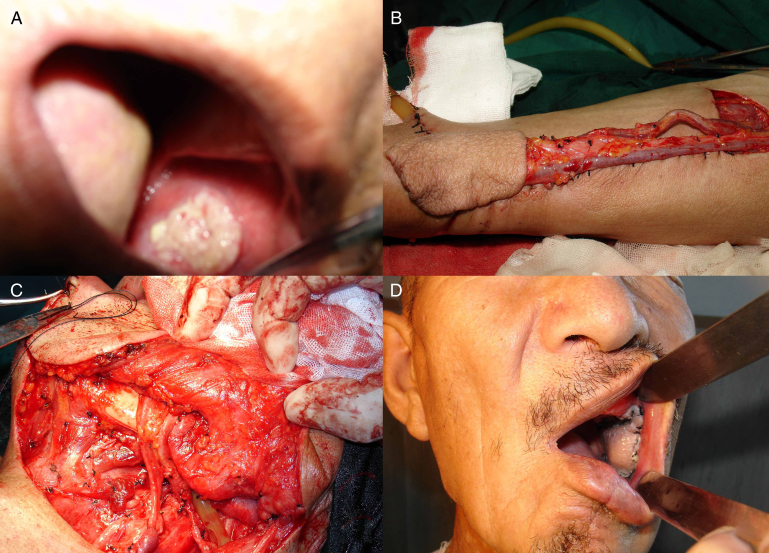
(A) Preoperative view of the buccal mucosa area; (B) the RF flap was harvested as the graft; (C) the flap was inset and anastomosed to the vessels; (D) postoperative result 1 month after operation.

A 60 year-old male patient had left buccal cancer (squamous cell carcinoma, stage T_2_N_1_M_0_). After excision of the tumor and radical neck dissection, the defect was reconstructed with a 6 cm × 4 cm RF flap.^11^ Anastomoses were performed to the facial artery, the retromandibular vein and the external jugular vein. The flap was healed uneventfully at the 3 year follow-up and satisfactory results were obtained (Fig. 2A–D).

#### Case 3

**Figure 3:**
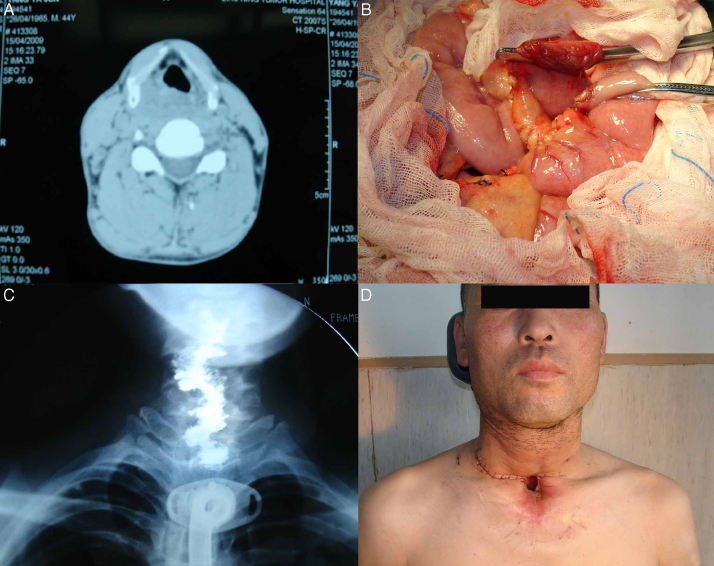
(A) Preoperative view of the hypopharyngeal cancer by CT; (B) the free jejunum flap was harvested from the left forearm; (C) barium swallow examination showing the postoperative result; (D) postoperative result 2 months after operation.

A 44 year-old male patient was diagnosed as squamous cell carcinoma of the hypopharynx (stage T_3_N_1_M_0_). Previous history included alcohol misuse. He had a total pharyngolaryngo-oesophagectomy (cervical faringolaringoesofagectomy) and free jejunal transfer. We harvested 12 cm of jejunum, which we transferred to the neck.^12^ Anastomoses were performed to the superior thyroid artery and the internal jugular vein. Two month after the operation, the patient was able to swallow a soft diet, and the result was satisfactory (Fig. 3A–D).

## Discussion

The strategy to treat head and neck cancer has mainly focused on surgery. However, this may lead to a large tissue defect and functional disability. Techniques in head and neck microvascular reconstruction not only have been advanced, but also have undergone a relative transformation in recent years. With limited donor site morbidity, high microvascular success rate, and decreased hospital stays, free flap reconstruction provides surgeons with opportunity to perform radical resections while minimizing cosmetic and functional defects.^14^, ^15^, ^16^ This retrospective review of 93 patients showed a success rate of 90.3%, which was in approximate agreement with other series with success rates exceeding 90%.^17^, ^18^, ^19^ In our study, all of the surgical procedures were performed by a single senior surgeon over a long period. Therefore, our surgery outcomes showed consistency by avoiding the influence of differences in the technical skill of multiple centers or multiple surgeons.

With the rapid development in microvascular reconstruction, the success rate of free flap is now very high. However, flap crisis and failure still occur, and the associated reasons are varied. In our study, the rate of surgical re-exploration to check microvascular anastomoses was 9.7%, and six of nine failed after re-exploration. We found venous thrombosis was nearly more than twice as common as arterial thrombosis and tended to develop later. It might be related to the rheology and biophysical properties of vein. Simultaneously, we noted that venous thrombosis behaved differently from arterial thrombosis. The former is the most commonly result of mechanical obstruction caused by twisting, kinking, stretching, and compression of veins. It is easier to manage, leading to a much higher salvage rate compared with arterial thrombosis. Based on our experience, most flap crises could be saved successfully within 3 h, but the probability of failure increases beyond this time. However, majority of the flap crises in this study were identified >8 h after surgery, and successful salvage rate was only 33.3%. Therefore, it is important to observe the flap. Monitoring the texture and capillary refilling of the free flap sometimes might be more effective than observing a color change. Doppler ultrasound might be not ideal, due to some interference from other vessels.

The analysis of the causes of flap crisis is essential to improving flap survival, which is fundamental to reconstruction. We analyzed the factors in our patients and their influence on the success rate. The findings did not indicate a significant correlation between flap failure and the patient's gender and age, this is supported by other authors,^20^, ^21^, ^22^ and the surgery can be regarded safe in elderly patients. Some authors revealed several factors like smoking, diabetes and vascular disease could affect the flap success rate.^23^, ^24^, ^25^ We demonstrated no significant influences of diabetes, hypertension, smoking and alcohol on flap necrosis. Because of the low rate of flap losses, our data did not allow us to affirm the role of them as risk factors. The impact of preoperative radiotherapy on free flap survival is a controversial topic, our results and the majority of reviewed studies did not show any significant relationship between both.^20^ In addition, some author suggested that the flap failure might be the result of technical error, injury to the vascular pedicle during harvest or poor recipient vessel selection.^26^ Lim et al. in their study found the patient's risk factors did not influence the outcomes of free flaps, and they concluded that the surgeon's expertise in performing microvascular surgical techniques was an important factor for the achievement of a good result.^27^ In contrast, Xu et al. suggested that the anastomosis technique was not likely to be the major cause of the flap crisis and the cause was likely to be unpredictable.^28^ In concordance with the previous literature, the surgeon's technique and experience have influence on flap success, and it should be laid enough emphasis in the clinical practice.

In our clinical experience, to facilitate microsurgical repair, the head can be turned slightly to the contralateral side. Double venous anastomoses might have a beneficial effect on survival of flaps compared with single venous drainage. Both the artery and specially the vein should be checked for either tension or redundancy before anastomosis. Except for visceral flaps such as jejunum, we routinely perform the arterial anastomosis first. The reason is that the completion of the arterial anastomosis allows the immediate evaluation of the venous return through the veins. A good return ensures the vein is orientated correctly with no twists. The patient's head is usually hyperextended and sometimes rotated to one side during reconstruction, and at that particular time, the pedicle may appear safely placed with the neck in this position, but once the head is returned to its normal position and the neck skin flap is pulled for final closure, the pedicle was instantly twisted or kinked. Thus, it is important to return the head and neck to a normal position first, sometimes with slight flexion, and then to inspect the placement of the vascular pedicle again before final closure. A drain can be placed in dependent portions of the wound, away from the site of microanastomoses, usually in the posterior triangle of the neck. After the surgery, the head should be maintained in a neutral position. Any mechanical compression in the neck must be strictly avoided.

Compromised flaps can be salvaged if problems are identified, but sometimes this can be difficult because the microcirculation often shuts down gradually.^29^, ^30^ The optimal strategy for managing complete flap loss is still subject to some debate at present. A failed free flap can be managed by one of three options: (1) a second free flap; (2) a pedicled flap; (3) conservative wound care followed by closure by secondary intention, skin graft, or delayed local flap. The choice between a second free flap or a pedicled flap might sometimes be difficult to make. In our study, the majority of patients were treated with conservative measures such as skin grafting, and only one patient used Pectoralis Major Myocutaneous flap. Currently, some studies suggested that in case of flap failure, the use of a second free flap was still more effective and reliable compared to a regional flap.^31^, ^32^ Although no patient received a second free flap following the first failure in this study, our practice was still guided by the principle: the strategy for managing a failed free flap should be firstly recognize and eliminate the underlying cause, then optimize of patient's general condition, reassess the defect to be reconstruction, and if necessary attempt a second free flap procedure.

In our institution, ALT and RF flaps are the work-horse flaps. In the US and Europe, the RF flap is more popularly used for the reconstruction of the orofacial region due to the reliability of its pedicle and the versatility in design.^33^, ^34^ However, it offers limited tissue supply, its complications are mainly related to the donor site, and it offers poor esthetics and morbidity. Since the ALT flap was first described in 1984,^35^ it has gained increasing popularity for a variety of defects, especially in Asia.^5^, ^10^, ^36^, ^37^ The ALT flap has two major advantages over the RF flap: a major artery is not killed and the scar can be easily hidden. The major problem with the ALT flap is the variation in the origin and course of supplying perforating branches of the descending branch of the lateral circumflex femoral artery. Simultaneously, in most European and North American patients, the lateral thigh donor site is considerably thicker than that of the Asian population due to the different body habitus and incidence of obesity.^38^ Thus, it has not gained widespread used in Europe and US. In our experience, both ALT flap and RF flap are reliable for the reconstruction. The ALT flap gives optimal results both at the donor site and the recipient site, and provides an ideal reconstructive option. The RF flap remains a valuable alternative in case of a thin soft tissue reconstruction due to its thinness and versatility.

The fibula flap is the most widely used osteocutaneous flap in China. It has some advantages, such as thick cortical bone around its entire circumference, making it suitable to withstand the forces of mastication and for the placement of dental implants. Because a large length of bone can be harvested, it is suitable for the restoration of subtotal and total mandibular defects.^39^ In our institute, the fibula donor site was used when a long segment of mandibular defect was to be bridged, particularly when these defects were anterior or lateral and include the ascending ramus. The jejunum flap remains a highly popular reconstructive option for circumferential pharyngeal defects or following total pharyngolaryngectomy. An abundance of jejunum can be harvested to reconstruct large defects. More importantly, swallowing is restored quickly, and 90% of patients can tolerate a regular or soft diet. Chan et al. reported the rate of postoperative fistula with jejunal flap was much lower than with ALT flap and RF flap.^40^ Our results were consistent with theirs: the fistula rate was 9.7% and 1.1% in patients with ALT flap and RF flap respectively, while no patient with jejunum flap had fistula. However, it is worth noting that the number of patients with jejunum flap is small, and much more patients from further studies are required to confirm our result.

In fact, the therapeutic goal for head and neck reconstruction nowadays might be not just filling a defect, but also the functional rehabilitation of the patient. In the present study, both ALT flap and RF flap could protect the original mouth-opening width well. The ALT flap tended to have a better result than RF flap. We deduced that the reason might be ALT flap has more soft tissue than RF flap, and the soft tissue could reduce fibrosis. There is evidence that speech and swallowing problems are related to tumor stage and location, and larger resections result in more functional problems in some studies. We found that patients with cancer of the tongue and the floor of the mouth had a poorer outcome than those with buccal tumors in terms of poor taste, swallowing and saliva. These results are similar to those found by Rogers et al.^41^ The presence of hair in the flap for men and an unacceptable scar in the donor area (especially for women) are minor disadvantages of the free flap. None of our male patients have complained about the hairy appearance of their flap. We think these disadvantages are not important when compared with the advantages of the flap.

There were limitations that could influence the result of our findings. First, our study was the retrospective analysis in a single-center study. Second, the sample size was small, and the number of patients with fibula myocutaneous flap and jejunum flap was also small. It may have resulted in limited statistical power. Another limitation to consider in this study was related to moderate skin laxity of the patients, because most patients were ≥50 years in this study and thus more likely to achieve good results in the defect closure than younger patients with mild skin laxity. Lastly, the median follow-up time of this study was rather short. Therefore, we look forward to obtaining more information from larger sample studies for a better comprehension of treatment and authenticating accuracy in a large population-based collective of patients in the near future.

## Conclusion

A wide range of reconstructive options are available for composite defects resulting from the treatment of head and neck cancer, the efficacy of which depends on the specific anatomy of the defect, planned outcome, the patient's tolerance for donor site morbidity, and the surgeon's training and experience. In general, the best option is the simplest one that will achieve all of the functional and esthetic goals of reconstruction. Our experience recorded in this study shows that, free tissue flap is a reliable and successful method of reconstruction in head and neck. However, surgeons should carefully assess and investigate the patients’ general health and coexisting conditions beforehand, and monitor flap perfusion carefully to improve the outcome and reduce the need for revision. This study should be further updated whenever new and strong evidence is available.

## Conflicts of interest

The authors declare no conflicts of interest.
